# The making of the Drosophila mushroom body

**DOI:** 10.3389/fphys.2023.1091248

**Published:** 2023-01-13

**Authors:** Suewei Lin

**Affiliations:** Institute of Molecular Biology, Academia Sinica, Taipei, Taiwan

**Keywords:** Drosophila, mushroom body, neural circuit assembly, neuronal remodeling, cell fate specification, axonal guidance, neural development

## Abstract

The mushroom body (MB) is a computational center in the *Drosophila* brain. The intricate neural circuits of the mushroom body enable it to store associative memories and process sensory and internal state information. The mushroom body is composed of diverse types of neurons that are precisely assembled during development. Tremendous efforts have been made to unravel the molecular and cellular mechanisms that build the mushroom body. However, we are still at the beginning of this challenging quest, with many key aspects of mushroom body assembly remaining unexplored. In this review, I provide an in-depth overview of our current understanding of mushroom body development and pertinent knowledge gaps.

## Introduction

The mushroom body (MB) comprises a pair of neuropils in the fly brain. It has conventionally been viewed as the olfactory learning and memory center, but recent studies have uncovered multifaceted roles for it in many other behaviors (Joiner et al., 2006; Pitman et al., 2006; Krashes et al., 2009; Keleman et al., 2012; Aso et al., 2014; Owald and Waddell, 2015; Lim et al., 2018; Tsao et al., 2018; Sayin et al., 2019; Senapati et al., 2019; Adel and Griffith, 2021). Development of the MB begins at early embryonic stages and continues until late pupal stages (Truman and Bate, 1988; Ito and Hotta, 1992). At early pupal stages, the larval MB is extensively remodeled into its more complex adult form (Armstrong et al., 1998; Lee et al., 1999; Truman et al., 2022). Accordingly, although still functional, the larval MB is simpler in terms of cell numbers and types than the adult MB (Aso et al., 2014; Eichler et al., 2017; Saumweber et al., 2018; Li et al., 2020).

### Adult fly mushroom body

**FIGURE 1:**
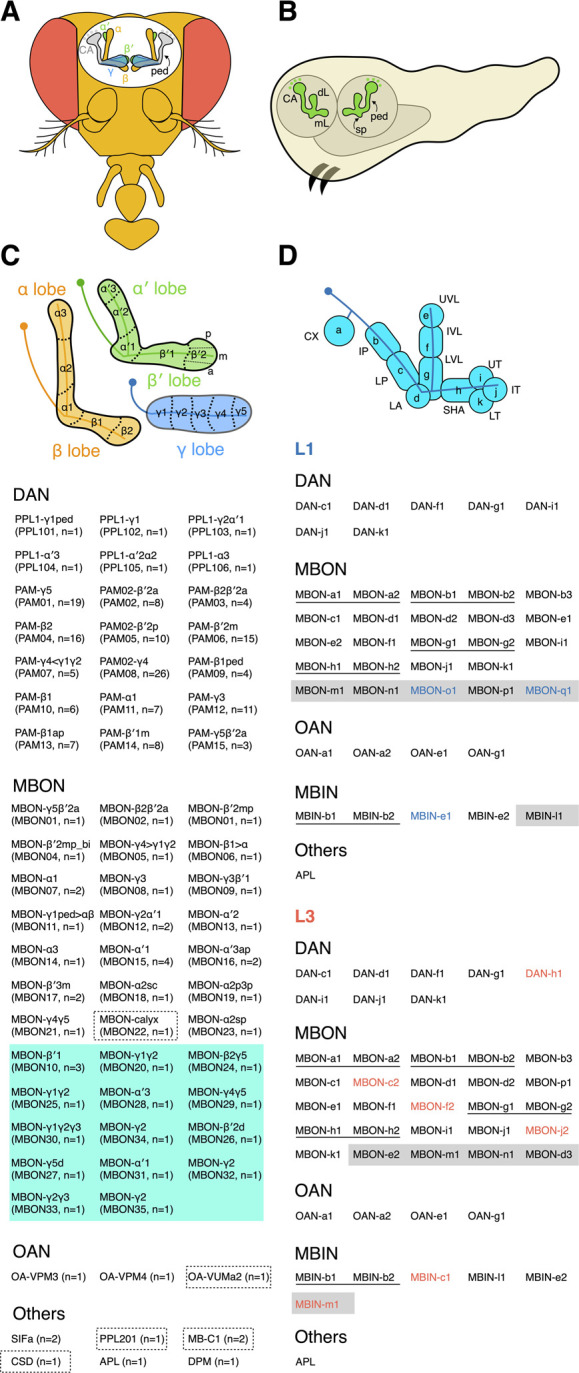
The adult and larval MB. **(A)** The adult MB has five lobes—γ, α, α′, β, and β′. The KC dendrites form a calyx (CA) near the cell bodies, and the peduncle (ped) connects the CA and lobes. **(B)** The larval MB has a dorsal lobe (dL), a medial lobe (mL), and a spur (sp) structure at the exit of the peduncle (ped). **(C)** The adult MB lobes are divided into 15 zones distinctively innervated by different extrinsic neurons. The β′2 zone can be subdivided into posterior (p), medial (m), and anterior (a) sub-zones. All MB extrinsic neuron types and their cell numbers are listed below the schematic. The inventory of extrinsic neurons is based on an electron microscopy (EM)-based reconstruction of the adult mushroom body (Li et al., 2020). Please refer to the same EM study for the morphology of each neuron. Both anatomical and numerical naming for MBONs and DANs are shown. The anatomical naming is based on the zones the neurons innervate. The atypical MBONs whose dendrites are not exclusively in the MB are grouped in a green-shaded area. The neurons primarily innervating the calyx are outlined with dashed boxes. **(D)** The larval MB is divided into 11 zones. Two different naming systems for the zones are indicated. The inventory of extrinsic neurons in L1 and L3 MBs is listed according to (Eichler et al., 2017; Saumweber et al., 2018). The neurons are named based on their zonal innervation, except for those (labeled with shaded areas) innervating multiple zones. Neurons of identical morphology (thus classified as the same type) are underlined. The neurons in blue or red font are found exclusively in the L1 or L3 MB, respectively.

The main framework of the adult MB comprises ∼2000 intrinsic neurons called Kenyon cells (KCs) (Aso et al., 2014; Li et al., 2020). The KC cell bodies are clustered in posterior regions of the brain, and their neurites are bundled together and extend anteriorly to form a stalk-like structure called the peduncle. At the end of the peduncle, the neurites separate to form three medial-projecting lobes (γ, β, and β′) and two vertical-projecting lobes (α and α′) (Figure 1A). The KC neurites constituting these MB lobes are considered axons, although they harbor both pre- and post-synaptic sites. The KCs are unipolar, and their dendrites branch out near the cell bodies to form a calyx structure.

The axons comprising the α and β lobes emanate from the same αβ KCs, whose neurites branch into one dorsal- and one medial-projecting process upon exiting the peduncle. Similarly, for the axons forming the α′ and β′ lobes, they are branches of the same α′β′ KCs. However, axons from the γ KCs do not have a dorsal branch and extend only medially to establish the γ lobe. These three main KC classes have been further subdivided into subtypes based on their morphologies and molecular markers (Tanaka et al., 2008; Aso et al., 2014; Li et al., 2020). For example, the γ KCs have dorsal (d) and main (m) subtypes, whose axons occupy distinct portions of the γ lobe; the α′β′ KCs have anterior-posterior 1 (ap1), ap2, and middle (m) subtypes, whereas the αβ KCs have posterior (p), surface (s), middle (m), and core (c) subtypes. See Aso et al., and Li et al., for the single-cell morphologies of these KC subtypes and lobe layers occupied by their axons (Aso et al., 2014; Li et al., 2020).

The MB lobes are densely innervated by extrinsic neurons. The two major extrinsic neuron classes are dopaminergic neurons (DANs) and MB output neurons (MBONs) (Tanaka et al., 2008; Aso et al., 2014; Li et al., 2020). The DANs project axons into the MB; they are the primary input neurons providing reinforcement and physiological state signals to the MB lobes (Waddell, 2013; Aso et al., 2014; Cohn et al., 2015; Owald and Waddell, 2015; Tsao et al., 2018; Senapati et al., 2019; Adel and Griffith, 2021). In contrast, the MBONs send dendrites to the MB lobes and are the primary output neurons relaying information from the MB to other brain regions (Tanaka et al., 2008; Aso et al., 2014; Li et al., 2020). At least 21 DAN types, 20 typical MBON types, and 14 atypical MBON types have been identified. Different types of DAN and MBON send axons or dendrites to arborize distinct regions in the MB lobes and subdivide them into 15 zones (Figure 1C). The typical MBON dendrites arborize almost exclusively in the MB lobes, whereas the atypical MBON dendrites also extend into the adjacent brain regions (Li et al., 2020). Each DAN and MBON type innervates one to three zones. These zones are the functional units of the MB, where DANs locally adjust the weight of the KC-to-MBON synaptic connections.

The MB lobes are also innervated by several other neuron types, including one GABAergic anterior posterior lateral neuron (APL), one serotonergic dorsal paired medial neuron (DPM), two SIFamide-expressing neurons (SIFa), and two octopamine-releasing neurons (OA-VPM3 and OA-VPM4) (Verleyen et al., 2004; Tanaka et al., 2008; Busch et al., 2009; Liu and Davis, 2009; Busch and Tanimoto, 2010; Lee et al., 2011; Aso et al., 2014; Li et al., 2020). Unlike the DANs and MBONs, these neurons do not exhibit zone-specific innervations. The neurites of APL and DPM neurons densely ramify the entire MB lobes and the peduncle, with the APL neurites also covering the calyx. The SIFa, OA-VPM3, and OA-VPM4 neurons only sparsely innervate the MB lobes (Aso et al., 2014; Li et al., 2020).

In addition to the APL neuron, the calyx is also occupied by several other cell types (Aso et al., 2014; Li et al., 2020). Both the composition and development of the calyx have been illuminated recently by an excellent review article (Punal et al., 2021). Thus, I do not discuss it further in this review.

### Larval mushroom body

The MB of the first instar larva (L1) contains only the embryonic-born γ KCs. Unlike their adult form, these KCs have bifurcated axonal branches. The dorsal-projecting branches form the larval dorsal lobe, whereas the medial-projecting branches form the medial lobe (Figure 1B). The L1 larval MB is innervated by seven DANs, 24 MBONs, 4 octopaminergic neurons (OANs), 1 APL neuron, and 5 additional modulatory neurons (MBINs) with unknown neurotransmitters (Eichler et al., 2017). The extrinsic neurons subdivide the L1 MB into 11 zones, including 8 in the lobes and 2 in the peduncle, as well as the calyx (Figure 1D).

As a larva grows, more KCs are incorporated into the MB, but the number and types of extrinsic neurons are mostly unchanging (Eichler et al., 2017; Saumweber et al., 2018). Specifically, the MB of third instar larvae (L3) has six additional extrinsic neurons, i.e., 1 DAN, 3 MBONs, and 2 MBINs (Figure 1D). In contrast, there are one MBINs and two MBONs found only in the L1 MB. These missing neurons could be due to cell death or the catalog of L3 MB neurons remains incomplete (Saumweber et al., 2018). In total, 44 extrinsic neurons have been identified in the L3 MB, including 8 DANs, 25 MBONs, 4 OANs, 6 MBINs, and 1 APL. The 44 extrinsic neurons are classified into 39 types based on their morphologies and molecular markers. Of these, 34 types have 1 cell in each hemisphere, except for OAN-a1 and OAN-a2 that have unpaired cell bodies at the midline in the maxillary and mandibular segments, respectively. The remaining five types have 2 cells in each hemisphere. Moreover, 38 out of the 44 extrinsic neurons innervate only one of the 11 MB zones. Of these 38 neurons, 14 also project to the contralateral MB, and all of them innervate the same zone on both sides. Six extrinsic neurons, including the APL, innervate multiple zones (Saumweber et al., 2018). Notably, the DPM neuron is not found in the L1 and L3 MB, indicating that this neuron type is adult-specific and only incorporated into the MB circuit during the pupal stage when the L3 MB is remodeled into the adult form (Eichler et al., 2017; Saumweber et al., 2018).

## Diversification of kenyon cells

### Embryonic stage

The KCs making up the MB in each hemibrain are produced by four neuroblasts (NBs) (Truman and Bate, 1988; Ito and Hotta, 1992). These NBs are the few in the fly brain that continue to divide and produce neurons throughout development, i.e., from early embryonic to late pupal stages. The postembryonic elements of the neuronal lineages produced by the four NBs are identical, but lineage-specific differences have been reported for the neurons generated during the embryonic stage (Kunz et al., 2012). Each MB-NB has a unique identity and is derived from a distinct progenitor in the procephalic neuroectoderm of the early embryo. Once MB-NBs have been specified, each of them expresses a distinct combination of transcription factors and thus is individually identifiable. The first 8–15 cells produced by MB-NBs are not KCs and they project neurites to other brain regions. The MB-NBs then switch to producing γ KCs, and the embryonic-born γ KCs derived from different MB-NBs differ in number and growth rate but eventually become morphologically indistinguishable in L1 larvae (Kunz et al., 2012). It is unclear if the γ KCs from different MB-NBs exert distinctive functions. The MB-NBs produce ∼95 γ KCs during the course of embryonic development (Kunz et al., 2012). This number is similar to the number of KCs remaining in the adult MB upon ablating MB-NBs in newly hatched hydroxyurea-fed larvae, indicating that all of the embryonic-born γ KCs survive into adulthood (Armstrong et al., 1998). Given that axons from these leftover KCs occupy the dorsal layer of the γ lobe, embryonic-born γ KCs appear to belong to the γd subtype (Armstrong et al., 1998). Consistently electronic microscopy-based reconstruction of the adult MB has uncovered 99 γd KCs (Li et al., 2020).

### Postembryonic stage

**FIGURE 2:**
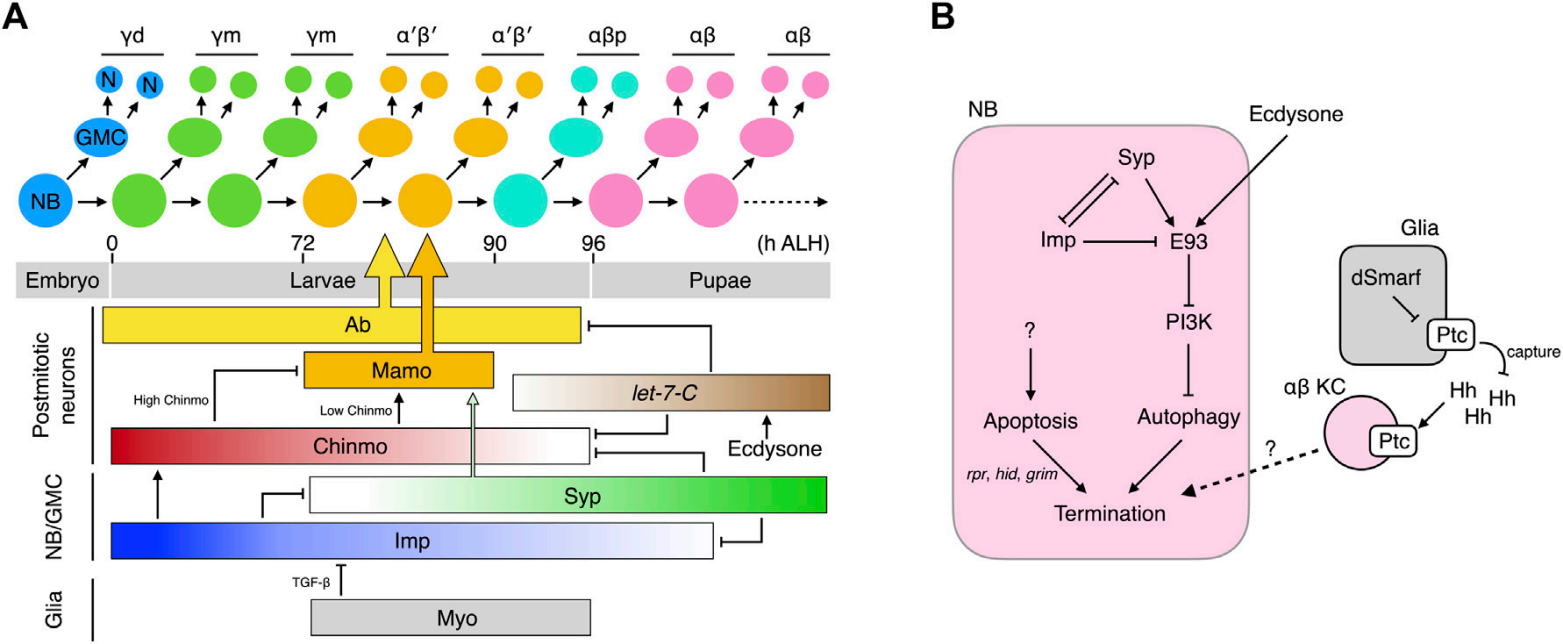
Temporal specification of KCs and termination of MB-NBs. **(A)** Production and birth order of KC types. The molecular network that specifies the KC types is shown. Ab and Mamo specifically instruct specification of the α′β′ KCs. They are regulated by multiple temporal factors that cooperate to also dictate specification of other KC types **(B)** Molecular networks that promote the termination of MB-NBs.

Unlike most other NBs that enter a quiescent state in newly hatched larvae, the MB-NBs continue to divide to make KCs (Truman and Bate, 1988; Ito and Hotta, 1992). In each division, a MB-NB produces a ganglion mother cell (GMC) and a self-renewed MB-NB (Lee et al., 1999). The GMC then divides once to produce 2 KCs (Figure 2A). The MB-NBs make different KC types at different developmental stages: all KCs generated from larval hatching to the mid-third instar become γ KCs (likely the γm subtype that occupies major parts of the γ lobe); KCs generated from the mid-third instar to ∼6 h before puparium formation become α′β′ KCs; KCs generated during the 6 h window before puparium formation become αβp KCs; and KCs generated during the pupal stage become αβ KCs (Lee et al., 1999; Zhu et al., 2006) (Figure 2A). The KC subtypes in each major class may also be produced sequentially during specific developmental time windows, though that supposition awaits experimental support.

How do the KCs born at different times acquire different cell fates? The first hint to the answer came from the discovery of *chinmo* (*chronologically inappropriate morphogenesis*) (Zhu et al., 2006). *Chinmo* encodes a BTB-zinc finger transcription factor, the protein concentration of which exhibits a high-to-low temporal gradient in newly-derived KCs from newly hatched larvae to those undergoing puparium formation. Chinmo dictates KC fates in a threshold-dependent manner: Chinmo^high^ specifies the γ fate; Chinmo^low^, α′β′; and Chinmo^none^, αβp. Altering Chinmo levels induces KCs to adopt inappropriate temporal cell fates. For example, increasing *chinmo* copy number makes KCs born after the mid-third instar adopt the earlier γ fate. In contrast, in a partial *chinmo* loss-of-function background, early-born KCs that normally become γ neurons instead adopt the later α′β′ or αβp fates (Zhu et al., 2006). The role of Chinmo in regulating the transition from αβp to pupal-born αβ KC is less obvious. If *chinmo* is completely deleted, the MB lacks γ and α′β′ KCs but hosts both αβp and pupal-born αβ KCs, suggesting that the αβp → pupal-born αβ transition can occur independently of *chinmo* (Zhu et al., 2006). However, strong overexpression of Chinmo makes all KCs adopt the γ fate (Zhu et al., 2006), and increasing the Chinmo level by removing its repressors *let-7* and *miR-125* delays the αβp → pupal-born αβ transition (Kucherenko et al., 2012; Wu et al., 2012). Thus, absence of Chinmo is necessary for specification of αβp and pupal-born αβ KCs, but additional mechanisms in which *let-7-C* plays a contributory role are required to promote the αβp → pupal-born αβ transition.

Several regulators have been unveiled as fine-tuning Chinmo levels, including the aforementioned *let-7* and *miR-125* transcribed from the *let-7-Complex* (*let-7-C*) locus (Kucherenko et al., 2012; Wu et al., 2012), as well as two RNA-binding proteins, i.e., IGF-II RNA-binding protein (Imp) and Syncrip (Syp) (Liu et al., 2015). The *let-7-C* miRNAs are negative Chinmo regulators and are expressed as low → high temporal gradients in postmitotic KCs during the time window when the α′β′ → αβp → αβ transitions are taking place (Wu et al., 2012). The *let-7-C* miRNAs lower Chinmo levels by binding to the 3′-UTR of *chinmo* mRNA and repressing its translation. Apart from *chinmo*, *let-7-C* also inhibits the expression of the transcription factor Abrupt (Ab) (Kucherenko et al., 2012). Ab is readily detected in the postmitotic KCs of L3 larvae when *let-7-C* levels are low, but its expression disappears in the pupal brain due to high levels of *let-7-C*. Ab promotes specification of α′β′ KCs, and its downregulation facilitates the specification of embryonic-born αβ KCs. Whether Ab also regulates the αβp fate remains undetermined. Taken together, the *let-7-C* miRNAs regulate α′β′ → αβp → αβ transitions by inhibiting the expression of two transcription factors, Chinmo and Ab.

In contrast to exclusive expression of Chinmo, Ab, and *let-7-C* miRNAs in postmitotic KCs, both transcripts and proteins of Imp and Syp are detectable in MB-NBs, in which they display opposing temporal gradients (Liu et al., 2015). Imp levels are high in L1 larvae and gradually decline until undetectable at 36 h after puparium formation (APF). In contrast, Syp levels are low in L1 larvae and gradually increase until at least 36 h APF. Imp and Syp reciprocally repress each other and contrastingly regulate *chinmo* translation; Imp promotes *chinmo* translation and keeps Chinmo levels high in early larvae, whereas Syp does the opposite and lowers Chinmo levels during late larval development.

In addition to Chinmo, both Imp and Syp also control other KC fate regulators. The range of the Imp temporal gradient is wider than that of Chinmo and extends into the early pupal stage. Knockdown of *imp* in MB-NBs results in the MB containing only pupal-born αβ KCs, which is phenotypically distinct from the *chinmo* mutant MB that comprises prominent αβp KCs in addition to pupal-born αβ KCs (Zhu et al., 2006; Liu et al., 2015). Therefore, low-level Imp may also promote the αβp → pupal-born αβ transition. Furthermore, Syp and Chinmo work cooperatively to control the expression of *maternal gene required for meiosis* (*mamo*), a BTB-zinc finger nuclear protein that specifies the α′β′ KCs (Liu et al., 2019). Low levels of Chinmo transcriptionally activate *mamo* expression during the temporal window when α′β′ KCs are born. High levels or lack of Chinmo both inhibit *mamo*, resulting in loss of the α′β′ KCs. However, low Chinmo is insufficient to drive an appropriate amount of Mamo, and Syp is also required post-transcriptionally to stabilize and promote maturation of the *mamo* mRNAs. Together, this multi-layered Chinmo/Syp control system ensures Mamo is expressed at the right time in the correct amounts. In addition to specifying the α′β′ KCs, Mamo has also recently been reported to promote and maintain the identity of the γ KCs during later development (Lai et al., 2022).

Apart from these intrinsic factors, KC fates are also regulated by extrinsic signals. Ecdysone signaling promotes the expression of *let-7-C*, and Myoglianin (Myo) from glia has been shown to act on its TGF-β/Activin type I receptor Baboon (Babo) in MB-NBs to promote specification of α′β′ KCs (Kucherenko et al., 2012; Marchetti and Tavosanis, 2019; Rossi and Desplan, 2020). Knockdown of *myo* in glia or of *babo* in MB-NBs leads to almost complete loss of α′β′ KCs without affecting the total number of KCs. The “lost” α′β′ KCs appear to be transformed into γ KCs (Rossi and Desplan, 2020) or αβp KCs (Marchetti and Tavosanis, 2019). Moreover, TGF-β signaling in MB-NBs helps to shape the Imp temporal gradient (Rossi and Desplan, 2020). Blocking TGF-β signaling elicits a higher Imp level that is more persistently above the threshold for specifying α′β′ KCs during the temporal window when these neurons are made. In contrast, expression of a constitutively active form of Babo in MB-NBs and their progeny expands production of α′β′ KCs (their percentage in total KCs increases from ∼26% to ∼32%). MBs with constitutively active TGF-β signaling still contain all KC types, unlike the γ KC-only MBs that arise from loss of Imp, indicating that additional timing mechanisms must contribute to regulating the Imp temporal gradient (Liu et al., 2015; Rossi and Desplan, 2020).

In summary, the KCs born during the postembryonic stage are temporally and sequentially specified into distinct cell fates. This process is coordinated by hierarchically-organized multi-layered intrinsic factors whose concentrations exhibit gradients during specific developmental windows. Extrinsic signals fine-tune the process by shaping the temporal gradients of these intrinsic factors (Figure 2A).

## Termination of KC neurogenesis

Unlike all other NBs that exit the cell cycle by ∼24 h APF, the MB-NBs continue to divide for 100 more cell cycles until ∼10 h before adult eclosion, when they are terminated by apoptosis and autophagy (Truman and Bate, 1988; Ito and Hotta, 1992; Siegrist et al., 2010). Early-pupal elimination of non-MB NBs is initiated by ecdysone signaling and the mediator complex (Homem et al., 2014). Prolonged Imp expression in the MB-NBs (relative to other NBs) of early pupae protects them from this early elimination process by inhibiting components in the mediator complex (Yang et al., 2017). At 72 h APF, the MB-NBs start to decrease in size, followed by a reduction in mitotic activity 6 h later and, finally, termination at 96 h APF (Siegrist et al., 2010). The reductions in MB-NB size and proliferation are caused by diminished PI3K signaling and subsequent nuclear entry of Foxo. The termination of MB-NBs is regulated by both apoptosis and autophagy. MB-NBs lacking proapoptotic genes (*reaper*, *hid*, and *grim*) survive to 3–5 days post-eclosion, but they are eventually eliminated. Blocking autophagy also slightly delays MB-NB termination. However, simultaneous blockage of autophagy and apoptosis dramatically extends MB-NB survival to 1 month in adulthood (Siegrist et al., 2010).

The molecular mechanisms underlying the activation of proapoptotic genes in MB-NBs remain poorly understood. The transcription factor Retinal homeobox (Rx) has been identified as a negative regulator of MB-NB apoptosis. Rx is expressed in both MB-NBs and MB-GMCs throughout the MB development and it loss leads to a premature loss of MB-NBs, which can be rescued by inhibiting apoptosis (Kraft et al., 2016). However, the precise relationship between Rx activity and apoptosis has not been established. Relatively more is known about the mechanisms regulating the autophagy of MB-NBs. It has been demonstrated that MB-NB autophagy is induced by Ecdysone-induced protein 93F (E93)-mediated downregulation of PI3K signaling (Pahl et al., 2019). E93 is a transcription factor expressed in the MB-NBs during later pupal stages (from 36 h APF). In the absence of E93, PI3K remains active in terminal-stage MB-NBs and thus inhibits autophagy, consequently prolonging their survival to the young adult stage. E93 expression is regulated by extrinsic ecdysone signal and the intrinsic temporal factors Imp and Syp. The ecdysone signal activates E93 expression; removing EcR from MB-NBs reduces E93 levels by half. Syp that is expressed at high levels during late pupal stages also positively regulates E93, and Imp prevents precocious E93 expression by inhibiting Syp expression during early development (Liu et al., 2015; Pahl et al., 2019). Notably, MB-NBs survive for at least 2 weeks into adulthood when Syp is knocked down, i.e., much longer than those lacking E93 or autophagy, which indicates that Syp exerts an additional role in MB-NB termination.

Glia has also been shown to regulate MB-NB termination (Yang et al., 2021). Elevating or reducing the Hedgehog (Hh) receptor Patched (Ptc) in glia suppresses or promotes αβ KC production, respectively. Ptc levels in glia regulate Hh signaling in KCs by modulating amounts of available Hh ligands. Glial Ptc is negatively regulated by dSmarf, a ubiquitin E3 ligase strongly expressed during pupal development when the αβ KCs are generated. Low Ptc in glia results in greater Hh availability to activate Hh signaling in the αβ KCs, which subsequently slows down MB-NB proliferation and promotes cell cycle exit through as yet unknown mechanisms. Therefore, as for KC fate specification, MB-NB termination is regulated by glial, hormonal, and intrinsic signals (Figure 2B).

## Branching, extension, guidance, and maintenance of the kenyon cell axons

**FIGURE 3:**
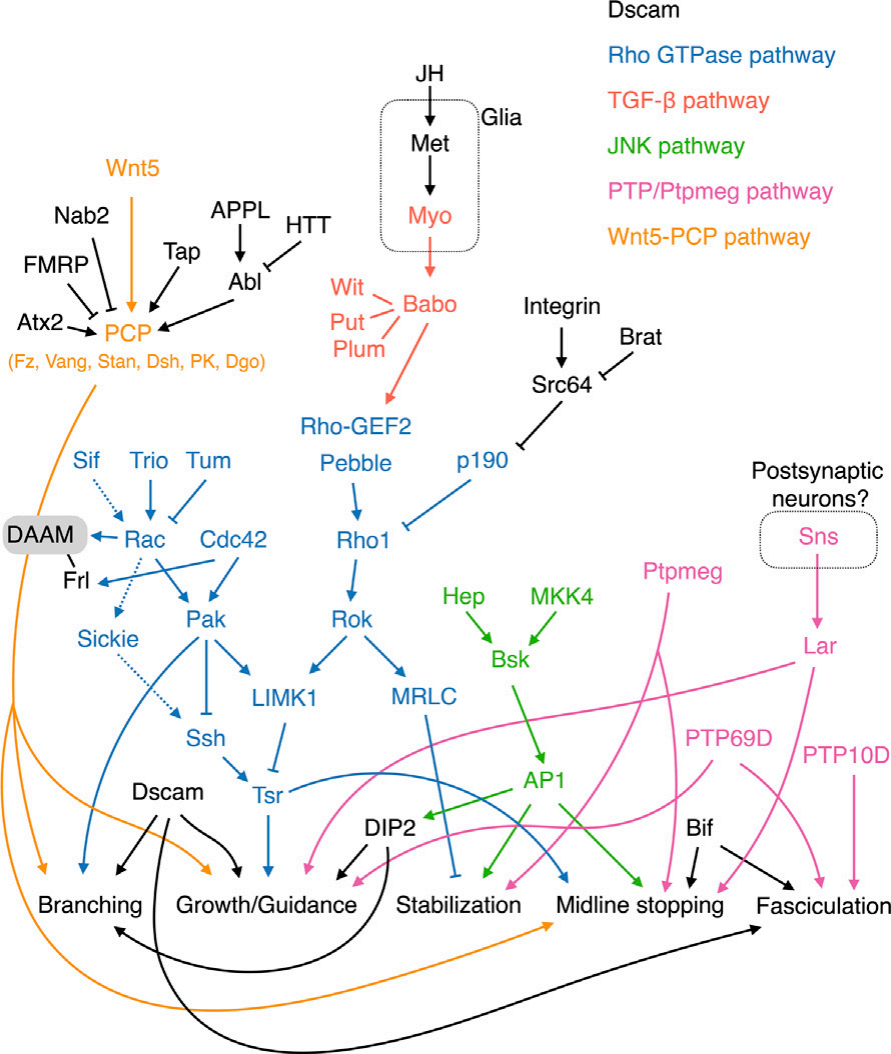
Molecular pathways regulating KC axonal development. The Wnt-PCP, Rho GTPase, TGF-β, JNK, and PTP/Ptpmeg pathways are colored orange, blue, red, green, and pink, respectively. Note that Tsr in the Rho GTPase pathway is phosphorylated by LIMK1 and dephosphorylated by Ssh. Cycles of Tsr phosphorylation (inactive form) and dephosphorylation (active form) are critical for axonal growth. The contrasting functions of Rac in activating and inhibiting Tsr are mediated by the Sif-Rac-Sickie (dashed arrows) and the Trio-Rac-Pak pathways, respectively. Molecules and pathways are assigned to functional categories based on their reported mutant phenotypes. The definitions for each category are as follows. Branching: Single KC mutant phenotypes are available, and the αβ or α′β′ mutant KCs exhibit either excessive or lack of sister branches. Growth/Guidance: Mutant phenotypes at the whole organism, whole MB, single MB-NB lineage, or single KC levels are available. Single mutant KCs exhibit short, misprojected, or unsegregated branches. When only a gross MB phenotype is available, it displays missing, truncated, or misprojected lobes. Stabilization: Mutant axons (at the whole MB or single-KC levels) exhibit normal morphologies at early developmental stages but are degenerated during later stages. Midline stopping: The mutant β or β′ lobes or branches overextend across the brain midline. Fasciculation: The axonal bundle of the mutant KCs shows splitting or defasciculation phenotypes when it travels through the peduncle. Note that the distinctions between these functional categories are not always clear-cut, and in some cases, the phenotypes assigned to different categories may only reflect different severities of defects in a common mechanism.

Many genes have been shown to regulate guidance and growth of KC axons (Table 1). However, a coherent overview of how these genes cooperate to regulate KC axons is still lacking. Here, I focus on some key genes and pathways in the regulatory network and describe how they contribute to our understanding of KC axonogenesis (Figure 3).

**TABLE 1:** Genes involved in the guidance and morphogenesis of KC axons.

Gene name	Functions	References
Down syndrome cell adhesion molecule (Dscam)	Br; Seg; Ex; Fas	Dong et al. (2022), Goyal et al. (2019), Hattori et al. (2009), Hattori et al. (2007), Hattori et al. (2008), Hong et al. (2021), Schmucker et al. (2000), Shi et al. (2007), Wang et al. (2004), Wang et al. (2002), Wojtowicz et al. (2004), Wojtowicz et al. (2007), Zhan et al. (2004)
**Rho GTPase pathway related**		
brain tumor (brat)	Stab	Marchetti et al. (2014)
Cell division cycle 42 (Cdc42)	AL-G	Ng and Luo, (2004)
LIM domain kinase 1 (LIMK1)	AL-G; βL-Stop	Ng and Luo, (2004), Ng, (2008)
Mig-2-like (Mtl)	Br; Ex; Gui	Ng et al. (2002)
p190 RhoGAP	Stab	Billuart et al. (2001)
p21-activated kinase (Pak)	AL-G	Ng and Luo, (2004)
pebble (pbl)	AL-G	Ng and Luo, (2004)
Rac1	Br; Ex; Gui	Ng et al. (2002)
Rac2	Br; Ex; Gui	Ng et al. (2002)
Rho guanine nucleotide exchange factor 2 (RhoGEF2)	AL-G	Ng and Luo, (2004)
Rho kinase (Rok). Also known as Drok	AL-G; βL-Stop	Billuart et al. (2001), Ng and Luo, (2004)
Rho1	Stab; βL-Stop	Billuart et al. (2001), Ng, (2008)
sickie (sick)	AL-G; Ex	Abe et al. (2015)
Slingshot (ssh)	AL-G	Ng and Luo, (2004)
Src oncogene at 64B (Src64)	αβL-G; βL-Stop; Stab	Billuart et al. (2001), Nicolai et al. (2003), Marchetti et al. (2014)
still life (sif)	AL-G	Ng and Luo, (2004)
Trio	AL-G	Awasaki et al. (2000), Ng and Luo, (2004)
tumbleweed (tum)	αβL-G; βL-Stop	Goldstein et al. (2005)
twinstar (tsr)	Ex; βL-Stop	Ng and Luo, (2004), Ng, (2008)
**TGF-β pathway related**		
baboon (babo)	βL-Stop	Ng, (2008)
Methoprene-tolerant (Met)	βL-Stop	Wu et al. (2021)
myoglianin (myo)	βL-Stop	Marmor-Kollet et al. (2019)
plum	βL-Stop	Marmor-Kollet et al. (2019)
punt (put)	βL-Stop	Ng, (2008)
wishful thinking (wit)	βL-Stop	Ng, (2008)
**JNK pathway related**		
basket (bsk)	αβL-G; Stab (all branches); βL-Stop	King et al. (2014), Nitta and Sugie, (2017a), Rallis et al. (2010)
DISCO interacting protein 2 (DIP2)	Br; Seg (mainly for αβ KCs)	Nitta and Sugie, (2017a, b), Nitta et al. (2017), Rallis et al. (2010)
hemipterous (hep)	Stab (all branches); βL-Stop	Rallis et al. (2010)
MAP kinase 4 (Mkk4)	βL-Stop	Rallis et al. (2010)
**PTP/Ptpmeg pathway**		
bifocal (bif)	βL-Stop; Fas	Kurusu and Zinn, (2008)
Leukocyte-antigen-related-like (Lar)	dL-G; βL-Stop	Bali et al. (2022), Kurusu and Zinn, (2008)
Protein tyrosine phosphatase 10D (Ptp10D)	Fas	Kurusu and Zinn, (2008)
Protein tyrosine phosphatase 69D (Ptp69D)	AL-G; Fas	Kurusu and Zinn, (2008)
Protein tyrosine phosphatase Meg (Ptpmeg)	Stab (α branch); βL-Stop	Whited et al. (2007)
Stick and stones (Sns)	AL-G; βL-Stop	Bali et al. (2022)
**Wnt5-PCP pathway related**		
Abl tyrosine kinase (Abl)	αβL-G	Marquilly et al. (2021), Soldano et al. (2013)
Ataxin-2 (Atx2)	βL-Stop	Rounds et al. (2022)
disheveled (dsh)	αβL-G; Br; Gui	Corgiat et al. (2022), Ng, (2012), Shimizu et al. (2011), Soldano et al. (2013), Yuan et al. (2016)
Fragile X messenger ribonucleoprotein 1 (FMRP)	αβL-G; βL-Stop; Br	Bienkowski et al. (2017), Michel et al. (2004), Pan et al. (2004)
frizzled (fz)	αβL-G; Br (preferentially control α branch)	Ng, (2012), Shimizu et al. (2011)
Huntingin (Htt)	αβL-G	Marquilly et al. (2021)
Nuclear polyadenosine RNA-binding 2 (Nab2)	αβL-G; βL-Stop	Corgiat et al. (2022), Kelly et al. (2016)
prickle (pk)	αβL-G	Ng, (2012)
starry night (stan)	αβL-G; Br	Shimizu et al. (2011)
target of pox (tap)	αL-G; βL-Stop	Yuan et al. (2016)
Van Gogh (Vang)	αβL-G; Br (preferentially control β branch)	Corgiat et al. (2022), Ng, (2012), Shimizu et al. (2011)
Wnt oncogene analog 5 (Wnt5)	αβL-G; Br; Seg	Grillenzoni et al. (2007), Shimizu et al. (2011)
β amyloid protein precursor-like (Appl)	αL-G; βL-G; β-Ex	Corgiat et al. (2022), Soldano et al. (2013)
**Others**		
Ankyrin-2 (Ank2)	AL-G	Siegenthaler et al. (2015)
castor (cas)	β/β′L-Stop	Hitier et al. (2001)
chickadee (chic)	Ex; βL-Stop	Ng and Luo, (2004), Worpenberg et al. (2021)
Cullin 3 (Cul3)	Ex; Fas	Zhu et al. (2005)
dachshund (Dac)	AL-G; Br	Kurusu et al. (2000), Martini and Davis, (2005), Martini et al. (2000), Noveen et al. (2000)
derailed (drl)	αβL-G; β/β′L-Stop; β/β′-Stop; Seg; Ex	Grillenzoni et al. (2007), Hitier et al. (2001), Moreau-Fauvarque et al. (1998), Simon et al. (1998)
derailed-2 (drl-2)	Seg; Gui	Reynaud et al. (2015)
Dishevelled Associated Activator of Morphogenesis (DAAM)	AL-G; Ex; Seg	Dollar et al. (2016), Foldi et al. (2022), Gombos et al. (2015)
Distal-less (Dll)	AL-G (based on larval MB phenotype)	Plavicki et al. (2012)
easily shocked (eas), also known as alpha lobe absent (ala)	Br; Seg; AL-G	Pascual et al. (2005), Pascual and Preat, (2001)
Edis	AL-G; βL-Stop	Xiong et al. (2022)
enabled (ena)	Ex	Ng and Luo, (2004)
Eph receptor tyrosine kinase (Eph)	Seg; αβL-G	Boyle et al. (2006)
Ephrin	αβL-G	Boyle et al. (2006)
eyeless (Ey)	AL-G (based on larval MB phenotype)	Callaerts et al. (2001), Kurusu et al. (2000), Noveen et al. (2000)
Fasciclin II (FasII)	Fas; βL-Stop; AL-G (based on larval MB phenotype)	Fushima and Tsujimura, (2007), Kurusu et al. (2002)
Forkhead box P (FoxP)	αβL-G	Castells-Nobau et al. (2019)
Formin-like (Frl)	Ex	Dollar et al. (2016)
frazzled (fra)	αβL-G	Kang et al. (2019)
glaikit (gkt)	Br	Nitta and Sugie, (2017b)
GTPase regulator associated with FAK (Graf)	βL-Stop; β-Stop	Kim et al. (2021)
Heterogeneous nuclear ribonucleoprotein at 27C (Hrb27C). Also known as hrp48	Br; Seg; αβL-G	Bruckert et al. (2015)
highwire (hiw)	αβL-G; Seg (for αβ KCs)	Shin and DiAntonio, (2011)
Histone deacetylase 4 (HDAC4)	αβL-G; βL-Stop	Main et al. (2021)
insomniac (inc)	αβL-G	Li et al. (2021)
Lipophorin receptors (LpR1 & LpR2)	αβL-G; βL-Stop	Rojo-Cortes et al. (2022)
Lysine demethylase 5 (Kdm5)	αβL-G; βL-Stop	Hatch et al. (2021)
Methyltransferase like 14 (Mettl14)	βL-Stop	Worpenberg et al. (2021)
Methyltransferase like 3 (Mettl3)	βL-Stop	Worpenberg et al. (2021)
miR-iab8-3p	αβL-G	Busto et al. (2016)
Moesin (Moe)	AL-G	Freymuth and Fitzsimons, (2017), Siegenthaler et al. (2015)
N-cadherin (CadN)	AL-G; Fas (based on larval MB phenotype)	Kurusu et al. (2012)
Nedd8 ubiquitin like modifier (Nedd8)	Ex	Zhu et al. (2005)
Netrin-B (NetB)	αβL-G	Kang et al. (2019)
Neuroglian (Nrg)	AL-G; Br; Ex; Gui; Fas	Carhan et al. (2005), Goossens et al. (2011), Siegenthaler et al. (2015)
partner of drosha (pasha)	βL-Stop	Luhur et al. (2014)
Plexin A (PlexA)	AL-G; βL-Stop	Shin and DiAntonio, (2011), Zwarts et al. (2016)
Plexin B (PlexB)	AL-G; βL-Stop	Zwarts et al. (2016)
polychaetoid (pyd)	αβL-G; βL-Stop	Goossens et al. (2011)
prospero (pros)	αβL-G; βL-Stop	Hatch et al. (2021)
roundabout (robo)	AL-G	Nicolas and Preat, (2005)
Semaphorin 1a (Sema1a)	AL-G; βL-Stop	Zwarts et al. (2016)
Tao	AL-G	King et al. (2011), King et al. (2014)
unc-5	αβL-G	Kang et al. (2019)
unc-51	Fas (based on larval MB phenotype)	Mochizuki et al. (2011)

unfulfilled (unf) YTH N6-methyladenosine RNA binding protein (Ythdf)	AL-G; Gui (however, Yaniv et al. suggest *unf* only has a role in γ axonal regrowth) βL-Stop	Bates et al. (2014), Bates et al. (2010), Lin et al. (2009), Yaniv et al. (2012) Worpenberg et al. (2021)

AL-G: required for axonal guidance or extension for all KCs (based on gross lobe morphology).

dL-G: required for guidance or extension of the dorsal lobes (based on gross lobe morphology).

αβL-G: required for axonal guidance or extension for αβ KCs (based on gross lobe morphology).

βL-Stop: required to prevent β lobe overextension and fusion (based on gross lobe morphology).

β-Stop: required to prevent β KCs from overextending across the midline (based on single-KC morphology).

β/β′L-Stop: required to prevent β and β′ lobe overextension and fusion (based on gross lobe morphology).

β/β′-Stop: required to prevent β and β′ KCs from overextending across the midline (based on single-KC morphology).

Stab: required to maintain axonal stability (based on lobe and single-KC morphologies).

Fas: required for axonal fasciculation (based on lobe and single-KC morphologies).

Br: required for axonal branching (based on lobe and single-KC morphologies).

Seg: required for segregation of axonal branches (based on lobe and single-KC morphologies).

Ex: required for axonal extension (based on lobe and single-KC morphologies).

Gui: required for axonal guidance (based on lobe and single-KC morphologies).

### Down’s syndrome cell adhesive molecule (Dscam)

*Dscam* encodes 38,016 alternatively-spliced cell surface proteins hosting 19,008 variable immunoglobulin (Ig) domains linked to one of two alternative transmembrane domains (Schmucker et al., 2000). The Ig domains exhibit strong isoform-specific homophilic interactions and have been proposed to provide each neuron with a unique molecular identity, allowing its sister neurites (expressing the same set of Dscam isoforms) to distinguish themselves from neurites of other neurons (expressing different sets of Dscam isoforms) (Wojtowicz et al., 2004; Hattori et al., 2007; Wojtowicz et al., 2007). The transmembrane domains regulate the distribution of Dscam in a sphingolipid-dependent manner (Wang et al., 2004; Shi et al., 2007; Goyal et al., 2019). Dscam proteins with a transmembrane domain encoded by exon 17.1 (TM1) or exon 17.2 (TM2) are selectively targeted to dendrites or axons, respectively (Wang et al., 2004; Shi et al., 2007).

*Dscam* is expressed in young KCs undergoing axonogenesis. Each KC expresses multiple *Dscam* isoforms, and different KCs express distinct sets of isoforms (Zhan et al., 2004). *Dscam* mutant αβ and α′β′ KCs exhibit axonal branch supremacy that often fail to segregate from each other, suggesting a role for *Dscam* in KC axonal branching and segregation (Wang et al., 2002; Zhan et al., 2004). Furthermore, *Dscam* mutant KC axons display a defasciculation phenotype upon parallel projection in the peduncle (Zhan et al., 2004). Interestingly, *Dscam* is not only required cell-autonomously to support segregation of KC axonal branches, but its diversity in the surrounding KCs is also essential (Zhan et al., 2004; Hattori et al., 2007; Hattori et al., 2009). It has been proposed that sister axonal branches from the same KCs recognize each other by expressing the same set of Dscam isoforms. The strong homophilic interaction between the same Dscam isoforms promotes self-repulsion that segregates the branches. These branches are not repelled from other KC axons, as these latter express different sets of Dscam isoforms. Moreover, the weak interactions between neurites expressing some of the same isoforms leads to adhesion, explaining the defasciculation phenotype observed in *Dscam* mutant KCs (Wojtowicz et al., 2004). This weak interaction-mediated adhesion may also help to “collapse” premature splitting in immature axons and suppress the branch supremacy phenotype observed in *Dscam* mutant KCs (Wang et al., 2002). In addition to the canonical role of Dscam in mediating self-avoidance and axonal fasciculation, recent studies have also revealed isoform-specific Dscam functions in KC axonal growth and guidance (Hong et al., 2021; Dong et al., 2022).

### Rho GTPase pathways

Rho family GTPases—including Rho, Rac, and Cdc42—are important signaling molecules that regulate axonal growth by modulating the cytoskeleton protein actin (Hodge and Ridley, 2016). Rho GTPase activity is tuned by upstream guanine exchange factors (GEFs) and GTPase activating proteins (GAPs), whose activities are regulated by signals transduced from surface receptors upon their binding to extracellular cues. Several actors in Rho GTPase pathways have been shown to play roles in various aspects of KC axonogenesis.

Rho GTPases cycle between active GTP-bound and inactive GDP-bound forms. GEFs activate Rho GTPases by catalyzing the exchange of GDP for GTP (Hodge and Ridley, 2016). Trio is a GEF strongly expressed in αβ KCs and weakly in γ KCs (Awasaki et al., 2000). The *trio* mutant MB exhibits severe growth defects in all lobes, suggesting that *trio* plays both direct and indirect roles in regulating KC axons. Trio has two GEF domains known to catalyze GDP-to-GTP exchange on Rho GTPases and that potentially interact with both Rho and Rac (Awasaki et al., 2000). Current evidence supports that Trio acts on Rac and its downstream effector LIM kinase I (LIMK1) to promote axonal growth (Ng and Luo, 2004).

GAPs negatively regulate Rho GTPases by converting them from the active GTP-bound form to the inactive GDP-bound form (Hodge and Ridley, 2016). The RhoGAP p190 is essential for maintaining the stability of KC dorsal branches (Billuart et al., 2001). Knocking down *p190* or weakly but constitutively activating Rho1 results in progressive retraction of KC dorsal branches. Rho1 regulates the stability of the KC dorsal branches *via* the downstream effectors *Drosophila* Rho-associated kinase (Drok) and myosin regulatory light chain (MRLC), which presumably mediate that function by modulating actin and myosin (Billuart et al., 2001). Furthermore, P190 is negatively regulated by integrin and its downstream tyrosine kinase Src64; loss of one copy of *Src64* or *myrospheroid* (*mys*) that encodes an integrin β subunit suppresses the *p190* RNAi phenotype. Consistently, loss of *brain tumor* (*brat*), which encodes a translational repressor of *Src64*, induces a maintenance deficit in KC axons (Marchetti et al., 2014). However, preventing *Src64* activity in KCs mainly causes overextension of the medial-projecting β branches, an outcome in stark contrast to the primary role of *p190* in the dorsal branches (Billuart et al., 2001; Nicolai et al., 2003). Also, the effect of *brat* loss-of-function is not restricted to dorsal branches (Marchetti et al., 2014). Therefore, the relationship between Src64 and P190 in regulating KC axonal maintenance remains to be elucidated. Notably, the Rho1 signaling that triggers retraction of KC dorsal branches is mostly dormant, with loss of Rho1 activity having no observable effect on KC axons (Lee et al., 2000b).

The fly genome encodes three Rac GTPases, i.e., Rac1, Rac2, and Mig-2-like (MTL) (Ng et al., 2002). All three of these Rac GTPases contribute to the development of KC axons. Combinations of mutant alleles for the three Rac GTPases elicit different degrees of abnormality in axonal branching, guidance, and growth. Axonal branching is the most sensitive to loss of Rac GTPase activity, followed by guidance and then growth. By expressing wild-type and mutant Rac1 capable of engaging different effector pathways, Ng and others demonstrated that Rac GTPases regulate KC axonal branching, guidance, and growth *via* distinct effector pathways (Ng et al., 2002). That study also reveals a community effect of KC axonal guidance and branching. When Rac GTPase activities are reduced in 1 KC lineage, all four lineages exhibit the same guidance or branching defect. However, the molecular mechanism underlying this community effect remains unclear.

Twinstar (Tsr), the fly homolog of human cofilin, is a key downstream effector in the Rho GTPase signaling pathway regulating KC axonal growth. Cofilin regulates the actin dynamics essential for growth cone mobility. KCs homozygous for *tsr* mutants exhibit profound axonal growth defects (Ng and Luo, 2004). Tsr activity is inhibited by LIMK1 that phosphorylates Tsr at serine 3, whereas Slingshot phosphatase (Ssh) activates Tsr by removing the phosphorylation moiety. Genetic interaction studies have provided evidence that LIMK1 is positively regulated by Rho1 and Drok, as well as by Rac1/Rac2/Cdc42 and their downstream effector p21-activated kinase (Pak) (Billuart et al., 2001; Ng and Luo, 2004). Thus, Rho1 is not only involved in regulating axonal stability but also axonal growth. In terms of this latter, Rho1 is also inhibited by P190 and, moreover, it is activated by two GEFs—Pebble (Pbl) and Rho-GEF2. Apart from inhibiting axonal growth *via* Pak and LIMK1, Rac1 and Rac2 also promote axonal growth through an alternative Pak-independent pathway. This alternative pathway is mediated by Sickie, a fly homolog of the microtubule-associated protein neuron navigator 2 in human, and Ssh that antagonizes the effect of LIMK1 on Tsr (Abe et al., 2015). The contrasting functions of Rac are differentially regulated by two Rac GEFs, i.e., Trio (for the Pak-dependent pathway) and Still life (Sif; for the Pak-independent pathway). Furthermore, the Rac GAP Tumbleweed (Tum) has been shown to limit KC axonal growth, possibly *via* the Pak-dependent pathway (Goldstein et al., 2005).

### TGF-β signaling

The Transforming growth factor β (TGF-β) pathway links extracellular cues and Rho GTPase signaling to control KC axonal growth. TGF-β receptors are complexes comprising type 1 and type 2 receptor serine/threonine kinases. Ligand binding induces trans-phosphorylation between type1 and type 2 receptors, which subsequently activates downstream Smad-dependent and -independent signaling pathways. Loss of the type 1 receptor-encoding gene *babo* from all KCs results in overextension of the β lobe across the brain midline, implicating TGF-β signaling in limiting the growth of β branches (Ng, 2008). Babo works together with the type 2 receptors Punt (Put) and Wishful thinking (Wit), as well as an accessory receptor Plum, to regulate β lobe growth (Ng, 2008; Marmor-Kollet et al., 2019). This regulatory mechanism acts independently of Smad but requires Rho GTPases/LIMK1/Tsr signaling.

The fly genome encodes three TGF-β ligands: Myoglianin (Myo), Dawdle (Daw), and Activin (Act). Knockdown of *myo* in glia but not in neurons causes ∼40% of β lobes to overgrow and cross the brain midline (Marmor-Kollet et al., 2019). Therefore, growth of the KC β branches appears to be limited by Myo secreted from glial cells. Whether Daw and Act also act as TGF-β ligands to regulate KC axonal growth has not yet been tested. It is conceivable that some glial cells located at the brain midline act as a source of Myo ligands, although direct evidence for that supposition is lacking. Notably, a juvenile hormone receptor, Methoprene-tolerant (Met), was recently shown to function in glia to control growth of β lobes, supporting potential cooperation between that juvenile hormone and TGF-β signaling to control KC axonogenesis (Wu et al., 2021).

### JNK signaling

Jun N-terminal kinase (JNK) signaling maintains KC axon stability. Unlike the Rho GTPase pathways that preferentially affect dorsal branches, loss of *basket* (*bsk*, which encodes JNK) results in progressive degradation of all KC axons (Rallis et al., 2010). Interestingly, a partial reduction of BSK activity induces β lobe overextension, indicating that different levels of JNK signaling regulate distinct aspects of KC axonal development. Consistently, it has been discovered that removing the activity of either of the two upstream JNK kinases—Hemipterous (Hep) and MAP kinase 4 (MKK4)—causes β lobe overextension, but depleting both of them results in KC axon destabilization (Rallis et al., 2010). BSK exerts its effects by phosphorylating the Activator protein-1 (AP-1) complex, which includes the transcription factors Jun and Fos as subunits. BSK activity levels are translated into graded AP-1 responses, with weak or strong AP-1 inactivation inducing β lobe overextension or KC axonal degradation, respectively. Apart from governing axonal growth and stability, BSK regulates guidance of αβ KC axons by controlling the expression of DISCO interacting protein 2 (DIP2) (Nitta and Sugie, 2017a).

### Receptor and cytoplasmic tyrosine phosphatases

Receptor tyrosine phosphatase (PTP) and the cytoplasmic tyrosine phosphatase Ptpmeg play diverse roles in regulating KC axonogenesis. Three PTPs—PTP10D, PTP69D, and Lar—are expressed in young KCs, and their loss of function leads to axonal fasciculation, extension, and guidance defects (Kurusu and Zinn, 2008). Deleting PTP10D and its neighboring gene *bifocal* (*bif*) together, but not either of them alone, causes defasciculation of the KC axonal bundles in the peduncle. This outcome indicates that these two genes function in the robust molecular machinery that promotes KC axonal fasciculation. Notably, *bif* mutant flies display a prominent β lobe fusion phenotype, supporting a role for Bif in preventing overextension of KC β branches. Specific removal of PTP69D from all KCs also causes axonal defasciculation, as well as axonal growth and guidance defects, evidencing its essential and pleiotropic role in KC morphogenesis. *Lar* mutant MBs do not display a defasciculation phenotype, instead presenting defects in the growth and guidance of the dorsal lobes. Additionally, loss of *lar* from KCs causes overextension of β branches. A recent study identified Sticks and stones (Sns), an immunoglobulin superfamily cell adhesion molecule, as a ligand for Lar (Bali et al., 2022). Pan-neuronal knockdown of *sns* phenocopies the *lar* mutant MB phenotype. Sns is not expressed in KCs and it may function in postsynaptic neurons to regulate KC axons. However, the exact source of Sns has not yet been identified. Thus, giving these research findings, PTPs work collectively to control multiple aspects of KC axonal development.

The MBs in *ptpmeg* ^*−/−*^ flies are morphologically similar to the *lar* mutant MBs, presenting thin dorsal lobes and fused β lobes (Whited et al., 2007). However, *ptpmeg* regulates KC development non-cell autonomously, and the thin dorsal lobe phenotype of the respective mutant flies has been attributed to axonal destabilization; the MB morphology is normal up to 36 h after pupation, but then the dorsal lobes become progressively degraded. The phosphatase activity of Ptpmeg is required to both stabilize the dorsal branches and prevent β branch overextension. However, the FERM domain in Ptpmeg is only necessary for dorsal lobe stabilization, but it is dispensable for limiting β lobe overgrowth. Thus, Ptpmeg likely regulates these two distinct features of KC axonogenesis *via* different effector pathways.

### Wnt-PCP pathway

At the heart of the *Drosophila* Wnt-Planar Cell Polarity (PCP) pathway are the Wnt5 ligand and the receptor complex consisting Frizzled (Fz), Van Gogh (Vang), and Starry Night (Stan). This pathway also involves the cytoplasmic transducer Disheveled (Dsh) and various adaptor or effector proteins, including Prickle (PK) and Diego (Dgo). Components in the Wnt-PCP pathway have been shown to regulate KC axonal branching, growth, and guidance through cell- and non-cell-autonomous mechanisms (Shimizu et al., 2011; Ng, 2012).

Fz, Stan, and Vang are expressed in young developing KCs (Shimizu et al., 2011). Mutant flies homozygous for *fz*, *Vang*, and *dsh* lose α, α′, and/or β lobes. Single-cell analysis indicates that the missing-lobe phenotypes are caused by various degrees of branching, extension, and guidance defects. Genetic interaction experiments also suggest that these genes work cooperatively to regulate KC axonal development. RNAi knockdown of *stan* and *pk* from KCs also elicits similar phenotypes as observed for *fz*, *Vang*, and *dsh* mutant flies (Shimizu et al., 2011; Ng, 2012). More detailed characterization of the PCP-associated mutant phenotypes revealed that *fz* ^*−/−*^ flies preferentially lose α branches, whereas *Vang* ^*−/−*^ flies preferentially lose β branches (Ng, 2012). These branch-specific effects imply α and β branches possess distinct PCP signaling configurations. RNAi knockdown and KC-specific rescue experiments have shown that these PCP-linked genes function in KCs. However, when their activities are abrogated in a small subset of KCs, the targeted KCs only display mild axonal growth defects. Conversely, overexpressing these genes in a subset of KCs in the mutant flies only partially rescued the axonal phenotypes in those KCs (Ng, 2012). Therefore, PCP signaling seems to function both cell-autonomously and through the neighboring KCs, so that a small number of mutant KC axons can be rectified by their wild-type counterparts.

Similarly to these observations for PCP-linked genes, *Wnt5* null flies exhibit severe MB lobe-missing defects that can be rescued by specific expression of wild-type *Wnt5* in KCs (Grillenzoni et al., 2007; Shimizu et al., 2011). However, when *Wnt5* is deleted from only one of the 4 KC lineages, that lineage develops normal-looking lobes, suggesting that WNT5 secreted from surrounding KCs is sufficient to support axonogenesis of the *Wnt5* ^*−/−*^ KCs in a non-cell-autonomous fashion. Consistently, it was found that overexpressing *Wnt5* in the KCs of a *Wnt5* null fly brain resulted in detectable WNT5 protein levels in brain regions around the KCs (Shimizu et al., 2011). Genetic interaction experiments have further demonstrated that one *Wnt5* hypomorph allele synergistically exacerbates the MB lobe defects in flies heterozygous for PCP-linked gene mutants (Shimizu et al., 2011). Thus, the Wnt-PCP pathway plays a pivotal role in regulating KC axonal development.

Several modulators are known to tune Wnt-PCP signaling in KCs. For example, Dsh is positively regulated by β amyloid protein precursor-like (APPL), the fly homolog of human APP, and Target of Pox (Tap), the fly homolog of neurogenin transcription factor. Consistent with the notion that Wnt-PCP signaling differentially controls the dorsal- and medial-projecting KC branches, APP supports β branch growth cell-autonomously, whereas it facilitates α branch growth non-cell-autonomously (Soldano et al., 2013). Single-cell *Appl* ^*−/−*^ αβ KC clones exhibit β branch growth defects but have normal α branches. In contrast, overexpression of a secreted but not a membrane-tethered form of APPL in those KCs rescues the α lobe growth defect. APPL regulates β branch growth by facilitating Abelson kinase (Abl)-dependent phosphorylation of Dsh (Soldano et al., 2013). Moreover, removing one copy of *Vang* significantly enhances the β branch but not the α branch defect in *Appl* ^*−/−*^ flies, consistent with Vang preferentially regulating β branch arborization and extension (Ng, 2012; Soldano et al., 2013). A recent study identified Huntingtin (HTT) as a negative regulator of Abl kinase activity, with diminished HTT activity suppressing the *Appl* mutant phenotype (Marquilly et al., 2021).

Tap regulates Dsh by enhancing its expression. Like APPL, Tap cell-autonomously regulates β branch extension but non-cell-autonomously supports α branch development (Yuan et al., 2016). However, in contrast to *Appl* ^*−/−*^ KCs that exhibit impaired β branch growth, *tap* ^*−/−*^ KC β branches often extend across the brain midline, manifesting as an overgrowth phenotype (Soldano et al., 2013; Yuan et al., 2016). Introducing one copy of the *dsh* null allele significantly enhances β branch overextension and defective α branch phenotypes in *tap* ^*+/-*^ flies (Yuan et al., 2016). This result is consistent with the notion that Tap regulates KC axonal growth *via* Dsh and further indicates that Dsh activity and expression may need to be delicately balanced to support β branch growth while simultaneously preventing its overgrowth.

RNA-binding protein Nab2 has been found to regulate the levels of PCP components. Comparing the proteomes of wild-type and *Nab2* homozygous mutant flies revealed that PCP pathway proteins, including Vang, APPL, and several putative PCP effectors, are differentially expressed in *Nab2* mutant flies (Corgiat et al., 2022). Nab2 is expressed in adult and pupal KCs (no data is available for larval KCs). The MBs in *Nab2* ^*−/−*^ flies display high penetrance of α lobe-missing and β lobe overextension phenotypes that can be rescued by specific expression of wild-type *Nab2* in the KCs (Kelly et al., 2016; Corgiat et al., 2022). Removing one copy of *Vang*, *Appl*, or *dsh* significantly rescues the *Nab2* homozygous mutant MB phenotypes, supporting that Nab2 regulates KC axonal development by tuning down PCP signaling (Corgiat et al., 2022). Genetic interaction experiments have also revealed that Nab2 works cooperatively with Fragile X messenger ribonucleoprotein 1 (FMRP) and antagonistically with Ataxin-2 (Atx2) to regulate α branch growth (Bienkowski et al., 2017; Rounds et al., 2022). FMRP and Atx2 are also RNA-binding proteins and thus may share target transcripts with Nab2.

Dishevelled Associated Activator of Morphogenesis (DAAM), a formin type of actin assembly factor, is a critical downstream effector of the PCP pathway involved in regulating KC axonal development (Gombos et al., 2015). *DAAM* mutant KCs exhibit various axonal growth and guidance defects. Genetic interactions between *DAAM* and PCP pathway genes have been observed. Reducing the activity of PCP pathway genes or *Wnt5* generally enhances *DAAM* mutant MB phenotypes. Furthermore, the *dsh* null mutation has no effect on the KC axonal defects caused by overexpressing a constitutively-active form of DAAM, supporting that DAAM works downstream of the PCP pathway (Gombos et al., 2015). Notably, *Rac1* heterozygosity strongly enhances the *DAAM* mutant MB lobe defects. Moreover, DAAM works cooperatively with Formin-like (Frl), another formin targeted by Cdc42, to regulate KC axonal development (Dollar et al., 2016). Together, these results support that DAAM/Frl may represent a convergence point for the PCP and Rho GTPase signaling pathways involved in KC axonogenesis.

## Remodeling of the mushroom body

The MB network undergoes dramatic reorganization to morph from the larval to the adult form during the pupal stage. Research on this remodeling process has revealed fundamental mechanisms underlying axonal pruning and regeneration. However, we have only begun to understand this complex process. In this section, I first focus on pruning of the γ KCs, the most studied MB remodeling process, and then move on to recent progress on other members of the MB circuit.

### Remodeling of the γ KC axons

The γ KC axons in larvae bifurcate upon reaching the end of the peduncle. One γ branch projects dorsally, and the other extends medially toward the midline, just like those of α′β′ and αβ KCs. The larval KC branches begin to degenerate right after puparium formation and, at 18 h into the pupal stage, they are completely trimmed back to their branching point. The γ KCs then regrow their axons, but this time only toward the midline and without the dorsal branch (Lee et al., 1999).

**FIGURE 4:**
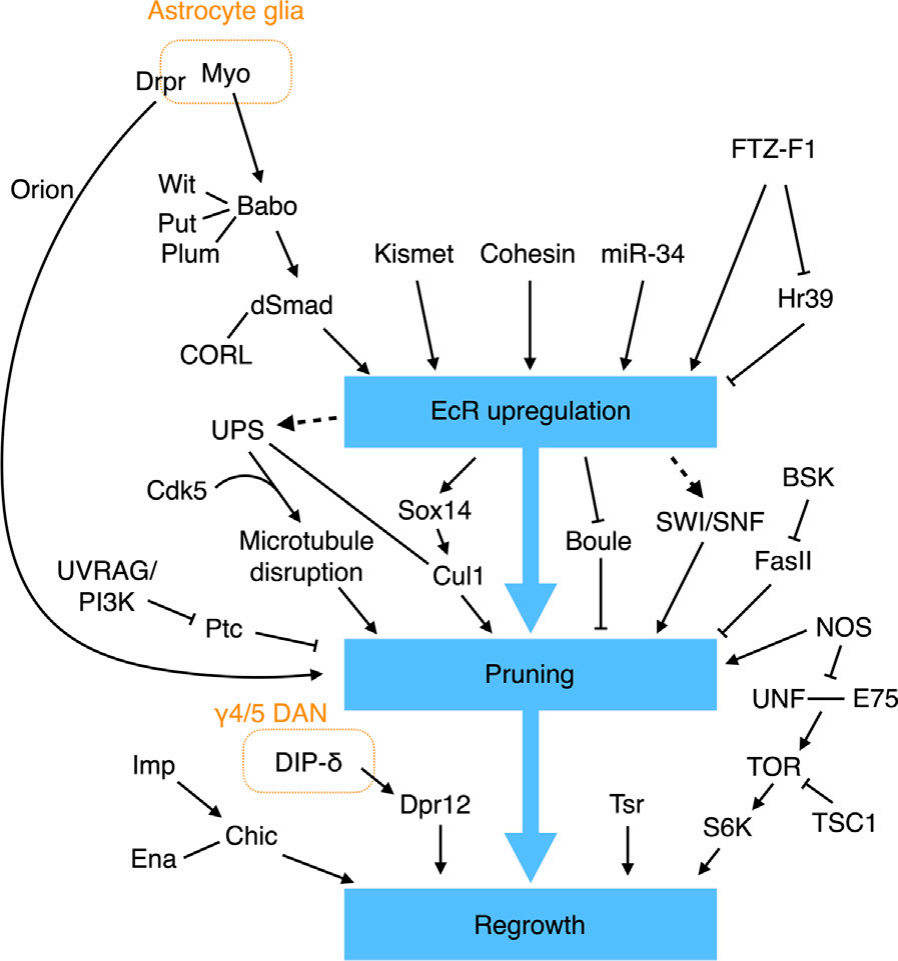
Molecular pathways regulating γ KC axonal pruning. EcR signaling plays a central role in regulating γ KC axonal pruning. EcR expression is regulated by multiple molecules and signaling pathways. Additional EcR-independent pathways have also been found to control γ KC axonal pruning and regrowth.

Studies over the past few decades have endowed us with an impressive understanding of the molecular networks that regulate pruning and regrowth of the γ KC axons (Figure 4). The first insights into the mechanism underpinning γ axonal pruning came from Lee and others, who identified mutations in *ultraspiracle* (*usp*) that block the pruning process (Lee et al., 2000a). USP and Ecdysone receptor B1 (EcR-B1) are nuclear receptors that form a heterodimeric receptor complex for ecdysone, a steroid hormone important for initiating molting and pupation (Thummel, 1996). EcR-B1 is highly expressed in γ but not α′β′ KCs, and removing EcR-B1 specifically from γ KCs prevents axonal pruning (Lee et al., 2000a). Thus, that study established an initial link between ecdysone signaling and γ KC pruning. Subsequent research has shown that the TGF-β pathway is essential for EcR-B1 expression in γ KCs. Removing the TGF-β type I receptor Babo or its downstream effectors dSmad2 and CORL from γ KCs significantly limits EcR-B1 expression and suppresses axonal pruning (Zheng et al., 2003; Takaesu et al., 2012). The TGF-β signaling responsible for upregulating EcR-B1 expression also requires two mutually redundant type II receptors, Wit and Put, and it is facilitated by Plum, an immunoglobulin superfamily protein (Zheng et al., 2003; Yu et al., 2013). Myo secreted from the cortex and astrocyte-like glia serves as the ligand to activate the TGF-β receptor in γ KCs (Awasaki et al., 2011). As mentioned above, Myo and TGF-β signaling also regulate KC cell fate specification and prevent overextension of β branches. Thus, glia and the TGF-β pathway play a central and multifunctional role in MB development.

EcR-B1 expression in γ KCs is also tuned by additional factors. The epigenetic factor Kismet binds to cis-regulatory elements in the EcR-B1 gene to promote histone methylation and acetylation, which are necessary to activate EcR-B1 expression (Latcheva et al., 2019). miR-34 (a microRNA) downregulates EcR-B1 expression, although the direct targets of miR-34 remain unclear (Lai et al., 2016). Cohesin, a complex that holds the sister chromatids together during mitosis, has also been shown to bind to the EcR locus to regulate EcR-B1 expression in postmitotic γ KCs (Misulovin et al., 2008; Pauli et al., 2008; Schuldiner et al., 2008). More specifically, loss of the cohesin subunit proteins SMC1 or Rad21 results in reduced EcR-B1 expression and γ KC pruning defects (Pauli et al., 2008; Schuldiner et al., 2008). Moreover, the regulatory cohesin subunit Stromalin (SA) appears to be necessary for γ KC axonal pruning (Schuldiner et al., 2008). Finally, the nuclear receptors FTZ-F1 and Hr39 contrastingly regulate EcR-B1 expression (Boulanger et al., 2011). FTZ-F1 activates EcR-B1 expression, whereas Hr39 suppresses it. FTZ-F1 also inhibits Hr39 expression. Deleting *Hr39* partially rescues the pruning defect caused by *ftz-f1* loss of function. Therefore, the activity of FTZ-F1 and its downregulating Hr39 is required for appropriate EcR-B1 expression and γ KC axonal pruning. How EcR-B1 signaling initiates γ KC pruning remains largely unclear, but it is achieved at least in part by upregulating the transcription factor Sox14 and Cullin-1 (Cul1, the core scaffold protein of the SCF E3 ubiquitin ligase), and by downregulating the RNA-binding protein Boule (Hoopfer et al., 2008; Kirilly et al., 2009; Wong et al., 2013). The SWI/SNF chromatin remodeling complex is also believed to work downstream of EcR-B1 to regulate γ KC pruning, but a direct link remains to be established (Chubak et al., 2019).

In addition to EcR signaling, the JNK pathway and nitric oxide (NO) also regulate axonal pruning of γ KCs. Reducing Bsk activity, a JNK in *Drosophila*, from γ KCs blocks their axonal pruning (Bornstein et al., 2015). Bsk kinase activity negatively regulates the stability and membrane localization of Fas II, with the pruning defects caused by loss of Bsk being a consequence of abnormally high levels of Fas II. Accordingly, weak cell-cell adhesion may be necessary for efficient axonal pruning. NO is an important signaling molecule in the nervous system. A high level of NO in γ KCs appears to promote axonal pruning (Rabinovich et al., 2016). Knockdown of NO synthase (NOS) specifically from γ KCs results in pruning defects, whereas overexpression of a constitutively-active NOS transgene leads to early pruning. The NOS activity is facilitated by the calcium sensor Calmodulin (CAM). Knockdown of *cam* from γ KCs reduces NO levels and phenocopies the *nos* mutant pruning defect. Notably, mutant flies homozygous for the α subunit of soluble guanylate cyclase (sGC) exhibit normal γ KC pruning activity, indicating that NO does not promote pruning *via* the canonical sGC pathway (Rabinovich et al., 2016). Finally, RNA profiling of the remodeling γ KCs has revealed several additional factors involved in pruning (Alyagor et al., 2018).

Several biological processes have been linked to γ KC axonal pruning, including selective disruption of microtubules, endosome-lysosomal degradation, and ubiquitin-mediated proteolysis. The earliest indication of γ KC axonal pruning activity is the appearance of blebbed axons, observed at 6 h APF (Watts et al., 2003). At 8 h APF, the axonal branches, but not the neurites in the peduncle, become fragmented. Starting from 12 h APF, the disconnected axonal fragments are gradually removed until very few remain at 18 h APF. Interestingly, selective disappearance of microtubules from the axonal branches, but not the peduncle, is evident at 8 h APF, i.e., before the fragmented axons are eliminated. Therefore, local destruction of microtubules in axonal branches represents one of the earliest steps in the axonal pruning process. Consistent with microtubules being destroyed, genes encoding components of the protein-degrading ubiquitin-proteosome system (UPS) are upregulated in γ KCs by ecdysone (Hoopfer et al., 2008). Consistently, it has been found that deleting from γ KCs *uba1* that encodes the ubiquitin-activating enzyme 1 (E1), the SCF E3 ligase core protein Cul1, or genes encoding proteasome subunits blocks axonal pruning (Watts et al., 2003; Wong et al., 2013). Genetic interaction experiments have also indicated that UPS cooperates with the neuronal cyclin-dependent kinase Cdk5 to drive microtubule degradation in γ KCs (Smith-Trunova et al., 2015). Furthermore, inhibiting EcR signaling in γ KCs blocks microtubule degradation, revealing that UPS may act downstream of the EcR pathway (Awasaki et al., 2006). In addition to UPS, the endosome-lysosomal pathway has also been shown to promote γ KC pruning by degrading the Hh receptor Ptc; this latter mediates an inhibitory signal that prevents pruning (Issman-Zecharya and Schuldiner, 2014).

Apart from releasing Myo to activate the TGF-β pathway, glia exerts another prominent role in γ KC pruning, i.e., engulfing the fragmented axonal debris. The first evidence linking glia to removal of the γ KC axons came from the observation that larval γ lobes are infiltrated by glial processes at 6 h APF when the pruning process begins (Awasaki and Ito, 2004; Watts et al., 2004). Lysosomal activity in glia is greatly enhanced during the pruning process, indicating that glia degrades the engulfed axonal fragments *via* the endosomal-lysosomal system (Watts et al., 2004). Subsequent studies have revealed that astrocyte glia are involved in this process, with its engulfment activity requiring two partially redundant pathways; the first pathway encompasses the scavenger-like receptor Draper (Drpr) and its adaptor Ced-6, and a second pathway includes Crk, Myoblast city (Mbc), and Ced-12 (Awasaki et al., 2006; Hoopfer et al., 2006; Hakim et al., 2014; Tasdemir-Yilmaz and Freeman, 2014). Notably, Drpr expression in astrocyte glia is regulated cell-autonomously by EcR signaling, so inhibiting EcR signaling in astrocyte glia severely delays Drpr expression and impairs axonal clearance (Hakim et al., 2014; Tasdemir-Yilmaz and Freeman, 2014). Thus, ecdysone acts on both γ KCs and astrocyte glia to drive axonal pruning, with glia playing a dual role in initiating and completing the pruning process. However, the ligand that activates Drpr to promote glial engulfment of the fragmented γ KC axons remains to be identified. Pretaporter (Prtp) and macroglobulin complement-related (Mcr) are two known Drpr ligands (Kuraishi et al., 2009; Lin et al., 2017), but *prtp* null mutant flies exhibit normal γ KC remodeling (Kuraishi et al., 2009), and any involvement of Mcr in γ KC remodeling has not yet been examined. A recent study identified a chemokine-like protein, Orion, secreted from pruned γ KC axons that drives infiltration and engulfment by astrocyte glia (Boulanger et al., 2021). However, given that the *Orion* mutant displays a stronger pruning defect than the *drpr* mutant, Orion may act on another as yet unidentified glial receptor.

After pruning, the γ KC axons regrow to make the adult γ lobe. This switch from pruning to regrowth is regulated by NOS activity. As mentioned earlier, high levels of NO promote γ KC axonal pruning. A time-course assessment of NO levels during the pruning process revealed that they are high when pruning begins and become low when regrowth is about to start (Rabinovich et al., 2016). This reduction in NO level is likely caused by enhanced production of a dominant-negative short NOS isoform (NOS-short) that inhibits NOS activity. High levels of NO inhibit γ KC axonal regrowth by interfering with dimerization of the nuclear receptors Unfulfilled (UNF) and ecdysone-inducing protein 75B (E75), which promotes γ KC axonal regrowth through the Target of rapamycin (TOR) pathway (Yaniv et al., 2012; Rabinovich et al., 2016). Thus, by contrastingly regulating pruning and regrowth, the NOS/NO system provides an elegant switching mechanism for these two mutually exclusive processes.

The RNA-binding protein Imp also regulates γ KC axonal regrowth. Imp is actively transported into axons during γ KC remodeling, and loss of Imp specifically blocks axonal regrowth without affecting initial growth of the larval branches (Medioni et al., 2014). Imp promotes axonal regrowth by directly binding to the 3′-UTR of *chickadee* (*chic*) mRNA to facilitate its axonal transportation. The *chic* mRNAs encode the fly homolog of the human actin-binding protein Profilin and they cooperate with the actin elongation factor *Ena* to support γ KC regrowth (Medioni et al., 2014; Yaniv et al., 2020). Notably, as described above, Imp also functions as a temporal factor to specify KC cell fates, highlighting its versatile role in instructing MB development.

Tsr, the fly homolog of the actin-severing protein cofilin in human, also plays a vital role in γ KC regrowth. Loss of *tsr* from γ KCs severely impairs initial growth of the larval branches and re-extension of the adult-specific axons (Sudarsanam et al., 2020). Microtubules fail to protrude into the filopodia-like structure at the tip of regrowing *tsr* ^*−/−*^ γ KC axons. This observation has prompted the hypothesis that loss of *tsr* results in an accumulation of F-actin, which obstructs microtubule protrusion, thereby impairing neurite growth. However, further experiments are needed to rigorously test that hypothesis.

Finally, a recent study elegantly revealed the importance of the interplay between γ KCs and the MB-projecting DANs in γ KC axonal regrowth (Bornstein et al., 2021). RNA profiling of the remodeling γ KCs has revealed that the family of defective proboscis response (Dpr) genes are strongly expressed during axonal regrowth (Alyagor et al., 2018; Bornstein et al., 2021). Among them, Dpr12 is enriched at the front end of the regrowing γ lobe. Moreover, in the adult γ lobe, Dpr12 specifically localizes in the γ4 and γ5 zones. *Dpr12* ^*−/−*^ KCs fail to fully extend their axons into the γ lobe and are curtailed at the proximal border of the γ4 zone. Remarkably, removing Dpr12’s binding partner DIP-δ from the DANs innervating the γ4/5 zones elicits mislocalization of Dpr12 and phenocopies the axonal extension defect of *dpr12* mutant KCs. These and subsequent experiments have established that Dpr12/DIP-δ cooperation promotes γ KC axonal regrowth and stabilizes inter-axonal connections between the γ4/5-projecting DANs and the adult γ KCs (Bornstein et al., 2021).

### Remodeling of the APL neuron

In each brain hemisphere, a single APL neuron extensively innervates both the larval and adult MB. The larval APL neuron has two major bifurcated neurite branches; one projects to and arborizes the entire calyx, and the other enters the dorsal lobe and arborizes to cover both the dorsal and medial lobes. At 6 h APF, when remodeling of the γ KCs begins, the APL neurite arborizes in the calyx and lobes also start to be pruned. The arbors, but not the two main branches, are almost completely removed at 12 h APF, and start to regrow at 18 h APF. The regrowth begins in the calyx but, unlike in the larval APL, the neurites of the adult APL are not restricted to the calyx but continue to grow and by 48 h APF they cover the peduncle. Re-arborization of the APL neurites in the MB lobes occurs after 48 h APF and eventually covers the entirety of the MB lobes by 72 h APF (Mayseless et al., 2018).

Limiting EcR signaling in APL neurons impairs pruning and, furthermore, specifically inhibits regrowth of the adult APL neurites into the γ lobe (Mayseless et al., 2018). APL neurons express both EcR-B1 and EcR-A isoforms at 0 h APF, but only EcR-A can be detected in APL neurons after 24 h APF. Therefore, pruning and regrowth of APL neurons may be regulated by different EcR isoforms. Although blocking APL neuronal remodeling has no effect on remodeling of the γ KCs, the opposite is true. Thus, if γ KC remodeling is blocked, APL pruning is hindered, leading to the growth of unpruned APL neurites into unpruned γ lobes in the adult MB. It is evident that γ KC axons, no matter whether regrown or unpruned, are capable of maintaining and attracting APL neurites. Inhibiting neuronal activity or synaptic release in unpruned γ KCs rescues the APL pruning defect. Moreover, inhibiting Ca^2+^/CaM signaling in APL neurons also rescues the APL pruning defect elicited by unpruned γ KCs. Therefore, synaptic transmission between the γ KCs and APL neurons appears to coordinate their remodeling process. Furthermore, enhancing adhesion between APL neurons and γ KCs inhibits pruning of both, consistent with the idea that downregulation of cell adhesion molecules is necessary for pruning (Bornstein et al., 2015). The ability of γ KCs to attract and maintain extrinsic MB neurons might be general, given that ectopic neurites from serotonergic, dopaminergic, and DPM neurons have also been observed to innervate unpruned γ lobes (Mayseless et al., 2018).

### Remodeling of the DANs and MBONs

The extrinsic neurons innervating the larval and adult MBs differ in number and morphology (Saumweber et al., 2018; Li et al., 2020). Do all these extrinsic larval neurons survive into adulthood? If yes, what are their adult counterparts? In a recent study, Truman and others tackled these challenging questions using a large collection of split-GAL4 lines and a conditional flip-switch strategy to permanently label specific L3 MB extrinsic neurons and follow their morphological changes throughout metamorphosis (Truman et al., 2022). All of the extrinsic neurons they followed underwent pruning, regrowth, or degeneration during metamorphosis and generally lost their arbors by 8 h APF, becoming completely pruned or exhibiting disrupted cell bodies by 18 h APF. The degenerated neurons then degraded into scattered debris by 24 h APF, with the remodeled neurons forming growth cones between 16 and 24 h APF so that most were completely remodeled by 48 h APF (Truman et al., 2022).

**FIGURE 5:**
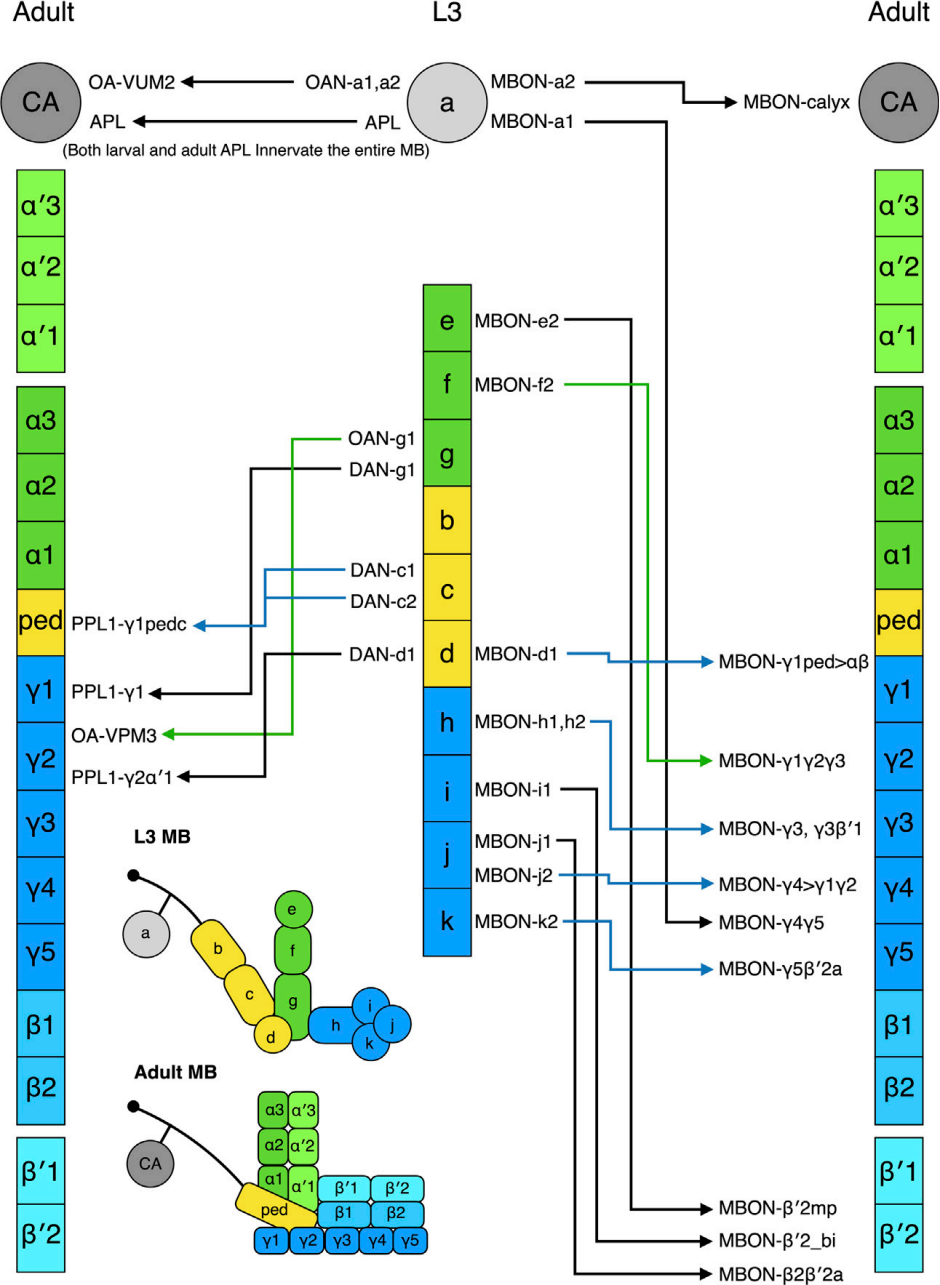
Metamorphosis of MB extrinsic neurons. The L3 MB extrinsic neurons that are remodeled into the adult MB extrinsic neurons are shown. The neurons have been positioned in front of the MB zones where their neurites innervate. Some neurons innervate multiple zones; in such cases, they have been placed in front of one of their innervating zones. The nomenclature of the neurons follows the definition provided in Figure 1.

The L3 MB has 8 DANs (8 types) and the adult MB has 157 DANs (21 types) innervating the lobes and one DAN arborizing in the calyx (Saumweber et al., 2018; Li et al., 2020). During metamorphosis, four larval DANs degenerate, and the other four are remodeled into adult DANs in the PPL1 cluster—PPL1-γ1pedc (2 cells), PPL1-γ1, and PPL1-γ2α′ (Truman et al., 2022) (Figure 5). The remaining adult DANs are produced post-embryonically and they are incorporated into the MB during pupal development. In contrast, most larval MBONs survive to adulthood. Of the 17 MBONs (13 types) that have been followed from larva to adult, none dies during metamorphosis, and 11 (9 types) are remodeled into 11 types of adult MBONs (Li et al., 2020; Truman et al., 2022) (Figure 5). These adult MBONs all project to the medial lobes, suggesting that all the vertical-projecting MBONs are adult-specific and added to the MB circuit during pupal development. Interestingly, larval MBON-a1 and MBON-a2 that are morphologically identical and classified as the same type morph into adult MBON-γ4γ5 and MBON-calyx that innervate distinct compartments of the adult MB. This pattern has also been observed for the identical larval MBON-h1 and MBON-h2, which become distinct MBON-γ3 and MBON- γ3β′1. Accordingly, morphology might not be an ideal criterion for classifying larval MBONs.

Strikingly, five larval MBONs (3 types) that innervate the larval vertical lobe or peduncle steer their neurites away from the MB during remodeling and innervate other regions of the adult brain. For example, larval MBON-b1 and -b2 transform into interneurons covering the adult lateral horn, whereas MBON-g1 and -g2 become neurons innervating the nodule in the adult central complex. The same “trans-differentiation” phenomenon also occurs in some larval OANs and MBINs, whose adult counterparts innervate various non-MB brain regions (Truman et al., 2022).

In summary, MB morphogenesis is accomplished by adding new neurons and remodeling the larval MB neurons. The adult DANs are mostly new additions, whereas about one-third of adult MBONs are transformed from their larval counterparts. The molecular mechanisms that coordinate remodeling and reassembly of the MB extrinsic neurons remain largely unclear. It would be interesting to test if the ecdysone pathway and Ca^2+^/CaM signaling that regulate remodeling of the γ KCs and APL neurons are also involved in remodeling other extrinsic neurons (Mayseless et al., 2018).

## Assembly of the MBONs and DANs

Compared to our knowledge of KC development, our understanding of how MB extrinsic neurons are assembled into the MB network is surprisingly limited. DANs and MBONs are the two main classes of MB extrinsic neurons. Their neurites arborize specific zones of the MB lobes. These zones have clearly defined borders, with minimal overlap of the axons and dendrites in the neighboring zones. Given that the MB lobes are bundles of continuous KC axons and that the DAN axons and MBON dendrites in each zone contact almost all the KCs in that zone (Takemura et al., 2017; Li et al., 2020), how these extrinsic neurites are organized represents an elaborate form of subcellular neurite targeting. How is such a compartmentalized organization established and what are the molecular and cellular mechanisms directing the innervation and arborization of the DAN axons and MBON dendrites? Answers to these intriguing questions are only beginning to emerge.

As indicated previously, the MBONs and DANs innervating the adult vertical lobes are mostly incorporated into the MB network during metamorphosis (Truman et al., 2022). Recently, my team followed the development of these extrinsic neurons during the pupal stage and found that their axons or dendrites sequentially innervate the MB zones in a stereotyped pattern (Lin et al., 2022). In general, innervation by the DAN axons initiates from the zones near the base of the vertical lobes and finishes at the tips of the vertical lobes, whereas the MBON dendrites innervate from the opposite direction, i.e., from the tips to the base. The innervation order for DAN axons and MBON dendrites projecting to the same zone varies. For example, the α3 zone is first innervated by the MBON dendrites, followed by the DAN axons, whereas the DAN axons precede the MBON dendrites in the α′2 zone.

This orderly innervation of the vertical lobes by MBON dendrites and DAN axons raises the possibility that the early-arriving neurites may provide guidance for subsequent ones. However, when early-arriving PPL1-α′2α2 DAN axons in the α′2 zone are ablated, targeting and arborization of later-arriving MBON-α′2 dendrites are not affected. Furthermore, ablation of MBON dendrites and DAN axons in one zone does not alter the dendritic and axonal innervations of the neighboring zones. Therefore, despite interactions among DANs and MBONs appearing to be a reasonable basis for establishing their zonal arborization patterns, their innervations in the MB lobes appear to be largely independent. Whether this scenario is also true for MBONs and DANs that innervate the horizontal lobes remains to be determined.

In contrast to the dispensable role of neighboring MBONs and DANs, KC axons are necessary for the correct arborization patterns of both MBON dendrites and DAN axons. In *alpha lobe absent* (*ala*) mutant flies in which the MB vertical lobes are absent, most MBON dendrites and DAN axons that normally target the vertical lobes wander around the missing lobes (Pascual and Preat, 2001; Pascual et al., 2005; Lin et al., 2022). Strikingly, when their usual target zone in the α′ lobe is missing, the MBON-α′2 dendrites steer away to innervate the β′2 zone of the horizontal lobes (a less prominent ectopic innervation is also found in an α′-like zone). The transmembrane guidance molecule Semaphorin 1a (Sema1a) functions as a receptor in MBON-α′2 to direct the ectopic innervation in the β′2 zone. However, Sema1a is dispensable for the MBON-α′2 dendrites to innervate their normal α′2 zone. These findings suggest that different MB lobe zones use distinct guidance signals to guide their innervation by MBON dendrites.

Overexpression of *sema1a* in the PPL-α′2α2 DAN misdirects its dendrites that reside outside of the MB lobes to the β′2, but also the α′1 and α′3, zones. Consistently, knockdown of *sema1a* in MBONs that normally project to these three zones results in significant dendritic innervation defects. Therefore, ligands for Sema1a likely work in combination with distinct guidance cues in each of the three zones to organize the zonal-specific innervation patterns of the MBON dendrites (Lin et al., 2022). Sema1a represents the first guidance molecule to be discovered that instructs zonal targeting of MBON dendrites. Identification of Sema1a ligands should provide insights into how the MB lobes are zonally patterned. Notably, the canonical ligands for Sema1a—Plexin A (PlexA), Sema2a, and Sema2b—do not appear to be required for MBON dendritic innervations (Cafferty et al., 2006; Sweeney et al., 2011; Jeong et al., 2012; Lin et al., 2022). No dendritic innervation defects have been observed for *sema2a/2b* double mutant flies or when *plexA* is knocked down pan-neuronally or in the glia. Consequently, it remains to be determined if these ligands function redundantly or if some non-canonical Sema1a ligands await discovery.

## Concluding remarks

The complex but highly modularized architecture of the MB underlies its ability to elicit a rich repertoire of behaviors in both larval and adult flies. Studying how the intricate MB architecture is built during development has provided profound insights into many neurodevelopmental processes, including temporal cell fate specification, axonal and dendritic guidance, neurite pruning and regeneration, neuron-glia interaction, and neural circuit morphogenesis. There is still much to learn from this beautiful structure in the fly brain. For example, we still know little about how the modular organization of the DAN axons and MBON dendrites is established in the MB lobes and what molecular signals instruct and coordinate remodeling of the entire network during metamorphosis. Given continuous advances in genetic labeling and manipulation techniques, long-term *in vivo* and *ex vivo* imaging, and single-cell RNA profiling and proteomics, a complete understanding of MB development is not out of reach.
